# Reduction of Dislocated Femur

**Published:** 1883-06

**Authors:** 


### Reduction of Dislocated Femur.

Dr. Samuel Logan, of New Orleans (*Gaillard's Med. Jour.*) re-asserts the plan proposed by him some years ago to reduce, by manipulation, luxations of the femur. He was induced to do this by the recital of the difficulties incurred and the means taken to overcome them in a case by Dr. C. Johnston, of Baltimore. This difficulty, mentioned by Mr. Callender, consists in the slipping of the head of the femur around the rim of the acetabulum during the process of the ordinary manipulative means for reduction, thus lodging the head of the femur either into the thyroid or sciatic-foramen, instead of into the acetabulum. This danger may be obviated, according to Dr. Logan, by making use of the anterior border of the pelvis as a fulcrum by which the head of the femur may be lifted over the rim of the acetabulum. When the thigh is fully flexed upon the abdomen, the limb, at the junction of its middle and upper third, impinges upon that portion of the pelvis just below the anterior superior spine of the ilium. Forced flexion now lifts the head of the femur, and rotation, outward for any form of posterior, and inward for any form of anterior dislocation, will throw the bone into place. An analagous idea has occurred to Dr. George Sutton, of Indiana, who suggested an artificial fulcrum of the arm of an assistant, or a roll of cloth placed in the groin. Both plans resemble the ancient method of placing a pillow between the thighs as a fulcrum for a similar purpose.

